# Maxillary sinus classification for sex and age using 23 artificial intelligence architectures

**DOI:** 10.1038/s41598-026-36112-1

**Published:** 2026-01-19

**Authors:** Wahaj Anees, Rianne Silva, Amber Khan, Jared Murray, Leonardo Scavassini, Mariana Burle, Nikolaos Angelakopoulos, Marcelo Henrique Napimoga, Lucas Porto, André Abade, Ademir Franco

**Affiliations:** 1 Division of Forensic Dentistry, Faculdade São Leopoldo Mandic, Campinas, São Paulo Brazil; 2 Dundee, Scotland, UK; 3 Department of Orthodontics and Dentofacial Orthopedics, University of Bern, Freiburgstrasse 7, 3010 Bern, Switzerland; 4 Division of Immunology, Faculdade São Leopoldo Mandic, Campinas, São Paulo Switzerland; 5 Computer vision and Engineering consultant, Brasília, Brazil; 6 Computer Science, Federal Institute of Science and Technology, Barra do Garças, Brazil; 7 Department of Therapeutic Stomatology, Sechenov University, Moscow, Russia

**Keywords:** Anatomy, Artificial intelligence, Convolutional neural networks, Forensic dentistry, Maxillary sinus, Sex, Computational biology and bioinformatics, Health care, Mathematics and computing, Medical research

## Abstract

Studies have relied on conventional imaging and traditional morphometric analyses of the maxillary sinuses (MS) for sex and age estimation, but little is known about the performance of deep learning models. This study aimed to evaluate the diagnostic accuracy of Convolutional Neural Networks (CNNs) and Vision Transformers (ViTs) in classifying individuals by sex and age through the radiographic assessment of the MS. Panoramic radiographs of individuals aged 6–22.99 years were sampled. Twenty-one CNNs and two Transformer-based architectures were tested. Tasks consisted of binary sex and age (≤ 15 vs. >15 years) and multiclass (sex + age) classifications. For sex classification, the highest accuracies were achieved by DeiT (0.807), ViT (0.806), and EfficientNetV2M (0.781), while for age classification, YOLOv11 (0.953), ViT (0.949), and DeiT (0.946) showed the best performance. The multiclass task yielded accuracies of 0.754, 0.753 and 0.734 by YOLOv11, DeiT, and ViT, respectively. Transformers consistently outperformed conventional CNNs, while YOLOv11 and EfficientNetV2M also demonstrated competitive performance. The studied artificial intelligence models may be useful as adjuncts for binary sex and age classification, but multiclass applications are still premature needing further research before their use in forensic practice can be recommended.

**Supplementary Information:**

The online version contains supplementary material available at 10.1038/s41598-026-36112-1.

## Introduction

The paranasal sinuses consists of cavities located in the maxillary, frontal, ethmoid and sphenoid bones^1^. Their functions are several^2^, including conditioning the air, supporting the immune system and reducing the weight of the skull. Hence, their importance is especially acknowledged in the fields of rhinology^3^, maxillofacial surgery^4^, immunology^5^ and dentistry^6^. Other specific applications fall within the field of forensic odontology^7^. The reasons are at least two: (I) because it is estimated that the paranasal sinuses may have a highly distinctive morphology, being potential features for antemortem/postmortem comparative human identification^8–10^; and (II) because the paranasal sinuses may be assessed to investigate differences between males and females (possibly yielding sex assessment)^11–13^, and their developmental timing (possibly yielding age assessment)^14,15^.

The maxillary (MS) are the largest paranasal sinuses^16^. These are the first to develop, around the 17th week of intrauterine life^17^. At this point, the MS is filled with amniotic fluid and is progressively aerated months or even years after birth^18^. Previous studies have suggested that there is a biphasic^19,20^ development of the MS that after birth, which is more accelerated during the first three years and after the age of seven. Additionally, the MS may increase until the end of puberty^21^; to reach its mature size around the age of 15 years or soon afterwards^22–24^. It is estimated that sex-related differences may occur after the age of eight^25^, being more expressive in the late teenagerhood^18,25^. Preliminary evidence indicates that MS are generally larger in males than in females^26^, suggesting that this morphological variation may serve as an exploratory parameter for forensic applications, particularly when integrated with artificial intelligence–based analytical approaches.

Studies on MS applications for sex and age assessment have been performed by means of bidimensional (2D) imaging, such as lateral cephalometric^27–29^ and panoramic^30^ radiographs, as well as by three-dimensional (3D) scanning, such as cone beam and multi-slice computed tomography^12^. Assessments have been performed through linear measurements, namely the height, width and length of the MS^11,24^, via the analysis of area and perimeter^28^, and calculating volume^11^. Studies on the MS morphology and inherent potential sex and age associations have expressed their findings in several ways, such as predictive equations^11^, correct classification rates^31^ and growth curves^32^.

Regarding the existing evidence in current scientific literature, a systematic review from 2023, screening 2475 individuals, demonstrated accuracy (acc) rates between 70 and 80% using the MS sinuses to assess sex by means of cone beam computed tomography^33^. A subsequent umbrella review on the maxillary, frontal and sphenoidal sinuses, revealed that the former was generally more dimorphic than the others, with acc rates around 70% when assessed via computed tomography^7^. Albeit not through a systematic review, authors have reported slightly lower acc rates for the analysis of the MS for age assessment^34^.

Computer-guided automation tools have been employed to study the MS, namely for 3D image segmentation^15^. However, the current body of knowledge lacks image-based investigations of the MS using deep learning and computer vision solutions, such as Convolutional Neural Networks (CNN) and Transformers-based architectures. CNNs are architectures designed to perform complex image pattern recognition^35^ – hence their recent application in sex and age assessment studies using radiographic samples^36–39^. Transformers are architectures originally developed to solve language-processing tasks, which are now being used as cost-effective alternatives to CNNs in terms of computational resources^40^. Coined as Vision Transformers (ViT), this solution has been tested for medical diagnostic purposes^41^, but not necessarily for forensic sex and age assessment via medical imaging. This is the gap to be addressed in the present study.

Based on the exposed, the current research aimed to perform the radiographic analysis of the MS challenging several CNNs and ViT models to sex and age assessment tasks in a diagnostic accuracy basis.

## Materials and methods

### Study design and ethical aspects

A diagnostic accuracy study was planned based on the performance of index tests (CNNs and ViTs) to classify children, adolescents and young adults according to sex and age. The classification task considered the recognition and analysis of images of the MS on panoramic radiographs. Medical images were retrospectively collected from an existing database. The images utilised in this study constituted secondary data sourced from an established radiology database (Center of Oral Radiology and Imaging). Access to the data was authorised through informed permission granted by the database’s legal custodian, and subsequently approved by the relevant ethics committee. No patient was exposed to ionizing radiation for research purposes since all the radiographs were acquired for clinical reasons. Ethical approval was obtained from the institutional committee of ethics in human research (protocol: 76809023.9.0000.5374). This study was reported partially following the *Standards for Reporting of Diagnostic Accuracy Studies Using Artificial Intelligence* (STARD-AI)^42^ and the key considerations for AI research articles in Dentistry^43^.

### Participants

The eligibility for sample collection considered as inclusion panoramic radiographs of Brazilian males and females from the Central-Western region, with age ranging from 6 to 22.99 years, with known date of birth and date of image acquisition. The radiographs were originally acquired between 2020 and 2025 and digitally stored. The exclusion criteria were images with surgical or orthopedic appliances in the maxillary region, piercings or any cosmetic products visible on the face, signs of skeletal malformation and radiographs with poor quality. The sample was structured into training, validation and testing set ups for each classification task (considering sex and age). To increase the sample size, right and left MS were combined as a single predictive morphological feature. Further dataset partitioning was performed at the participant level, meaning that only one image from each patient was assigned to a single split and not to another (i.e. train or validation), thereby preventing data leakage and promoting unbiased evaluation of model generalization. The selected images were analyzed in a Dell Inspiron 5590 (Dell Technologies Inc., Round Rock, Texas, USA) for annotations.

### Analysis

**Fig. 1:**
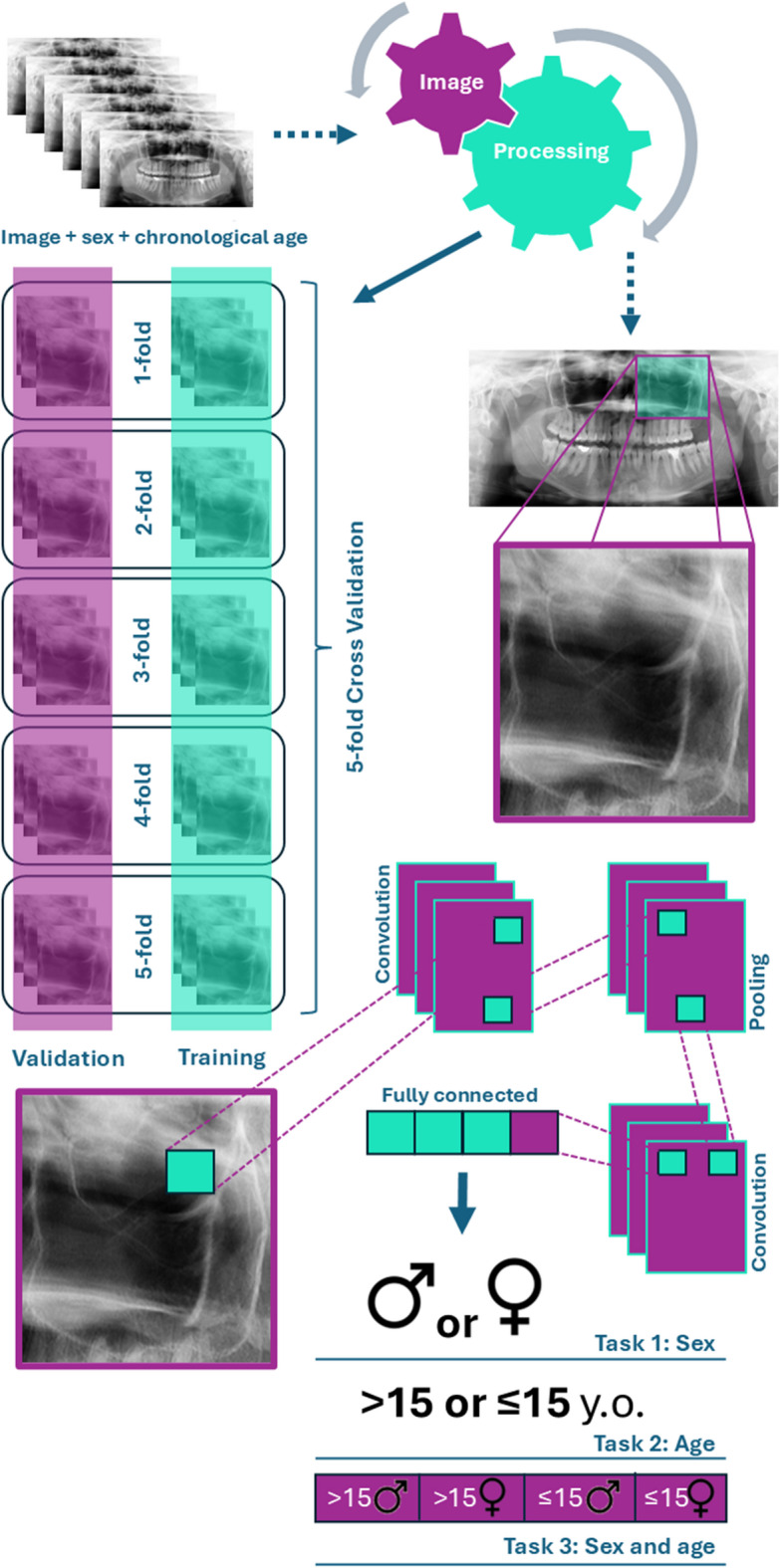
Research workflow displaying the dataset, image processing, annotation of the region of interest, cross-validation, convolution, and binary/multiclass classifications.

In a pre-processing phase, the images were anonymized and the radiographic identification of image side (left/right) was cropped out. Further patient identification was restricted to an alphanumeric code. To promote higher standardization, the images were also pre-processed preserving their size, image detail, spatial resolution, and quality. Within Darwin V7 software package (Darwin V7 Labs, London, UK), image annotation was conducted. To this end, the bounding-box tool was used. This tool enabled the selection of the region of interest using a rectangular frame that was manually dragged over the left and right MS (Fig. 1). Annotations were performed by five trained forensic odontologists with experience in the analysis of panoramic radiographs^36–39^, followed by a quality check by a supervisor forensic odontologist with 13 years of experience in research and practice.

Considering the MS as the region of interest (ROI), a bounding box margin of 8–10% was adopted to preserve the MS contour and best fit its anatomic context. The images were resized to 224 × 224 pixels, converted to 3 channels (replication of the grayscale), scaled to the [0,1] range, and min–max normalized per image. Image augmentation was applied to the training dataset and included the following transformations with the indicated probabilities: random horizontal flip (*p* = 0.5), rotation of ± 7° (*p* = 0.5), translation of ± 6% (*p* = 0.3), zoom between 0.9 and 1.1 (*p* = 0.3), brightness/contrast variation of ± 10% (*p* = 0.3), Gaussian noise with σ = 0.01 (*p* = 0.2) and mild sharpening (*p* = 0.2). A comparative deep learning approach based on 23 architectures and 100 epochs each was performed. Twenty-one CNN-based architectures were used: DenseNet121^44^ , DenseNet169^44^ , DenseNet201^44^, VGG16^45^, VGG19^45^, ResNet50^46^, ResNet50V2^47^, ResNet101^46^, ResNet101V2^47^, ResNet152^46^ , ResNet152V2^47^, InceptionV3^48^, Xception^49^, InceptionResNetV2^50^, NASNetLarge^51^, MobileNetV2^52^, MobileNetV3Large^53^, EfficientNetV2B0^54^ , EfficientNetV2M^54^, an ensembled architecture of two CNNs (established per task), and YOLOv11^55^. Additionally, two transformers-based architectures were employed: ViT^40^ and Data-efficient Image Transformer (DeiT)^56^.

Categorical cross-entropy loss and L2 regularization (weight decay = 5 × 10⁻⁴) was used in the training, implemented in the stochastic gradient descent optimizer with a learning rate of 0.0125, momentum of 0.937, 100 epochs, and a batch size of 16. Model evaluation was performed with 5-fold cross-validation^57,58^ , where in each iteration approximately 20% of the images were retained as an external test set, while from the remaining ~ 80%, about 10% was reserved exclusively for monitoring the training process. Early stopping was not applied; instead, at the end of 100 epochs, the checkpoint with the lowest monitoring loss was selected and subsequently evaluated on the corresponding test fold. We reported the average performance across all five folds. The choice of k = 5 represented a balance between computational cost and robustness: increasing k linearly raises the training cost (e.g., k = 10 would double the computational burden without proportionally improving precision), while with our sample size each fold provided a sufficiently large test set to yield stable estimates and a training set large enough to preserve generalization. This arrangement ensured that all images were used once as test data, allowed confidence interval estimation from the distribution of fold scores, and provided a practically robust yet computationally feasible evaluation strategy. Two experienced engineers conducted the computer-vision analytical process.

### Test methods

The architectures’ performance were tested for three tasks: [binary] sex-classification task, [binary] age-classification task, and [4-category multiclass] sex-age-classification task. Binary classifications considered the decision cutoff of 0.5, while multiclass task decisions considered the highest predicted probability. For the sex estimation task, the total number of unique images was 18,767, being 16,889 partially used for training and validation, and 1,878 for (external) testing. Class-wise, this included 10,002 females and 8765 males. For the binary age estimation task, the number of unique images was 18,754, being 16,877 for training and validation, and 1877 for testing. Class-wise, this task included 9942 individuals aged ≤ 15 years and 8824 individuals aged > 15 years. Finally, the combined sex and age multiclassification task totaled 18,767 unique radiographs, being 16,888 for training and validation, and 1879 for testing. Small differences between the total number of unique images across the three tasks as justified by the task-specific eligibility criteria. Specifically, each task was built from the subset of images with complete and valid labels required for that prediction problem (sex, age, or both). Hence, during dataset curation, quality-control filters (e.g., unreadable/corrupted files, duplicates/near-duplicates, and label inconsistencies/out-of-range values) were applied leading to slightly different numbers of unique images (e.g., 18,767 vs. 18,754), even though the acquisition source was the same. A consolidated overview of the sample size distributed per task, phase (training, validation, testing) and cross-validation folds is presented in Table 1.

**Table 1:** Sample distribution per task, study phase (training, validation and testing) and cross-validation folds.

Task	Fold	Training	Validation	Testing
Sex Unique: 18,767 Training and validation: 16,889 External test: 1878	1	13,511	3378	1878
	2	13,511	3378	1878
	3	13,511	3378	1878
	4	13,511	3378	1878
	5	13,512	3377	1878
Age Unique: 18,754 Training and validation: 16,877 External test: 1877	1	13,501	3376	1877
	2	13,501	3376	1877
	3	13,502	3375	1877
	4	13,502	3375	1877
	5	13,502	3375	1877
Sex and age Unique: 18,767 Training and validation: 16,888 External test: 1879	1	13,510	3378	1879
	2	13,510	3378	1879
	3	13,510	3378	1879
	4	13,511	3377	1879
	5	13,511	3377	1879

As reference standards, the architectures’ performance during the classification tasks was compared with individuals’ documental sex (male or female) and chronological age. The diagnostic accuracy performance metrics of the architectures using the MS to classify individuals were loss, acc, F1 score, precision, recall, and specificity. To account for the variability across different subsets of the data, mitigate overfitting ensuring that the performance metrics are not biased towards a specific part of the dataset, and to reach an overall performance of the model, this study calculated the average of each metric across all five folds. The outcomes were presented by means of confusion matrix and Receiver Operating Characteristic (ROC) curves and their area under the curve (AUC). Moreover, a visual pairwise comparison was enabled by using heatmaps of acc differences between architectures and expressing statistical significance after bootstrap analysis. Also in this context, a circular network based on architecture superiority was presented for each task showing how the models statistically outperformed others. Computer processing was performed with a Linux machine, with Ubuntu 20.04, an Intel^®^ Core^™^ i7-6800 K processor, 2 Nvidia^™^ GTX Titan Xp 12 GB GPUs, and 64 GB of DDR4 RAM. All models were developed using TensorFlow API^59^ version 2.18. Python 3.8.10 was used for algorithm implementation and data wrangling^60^.

## Results

**Fig. 2:**
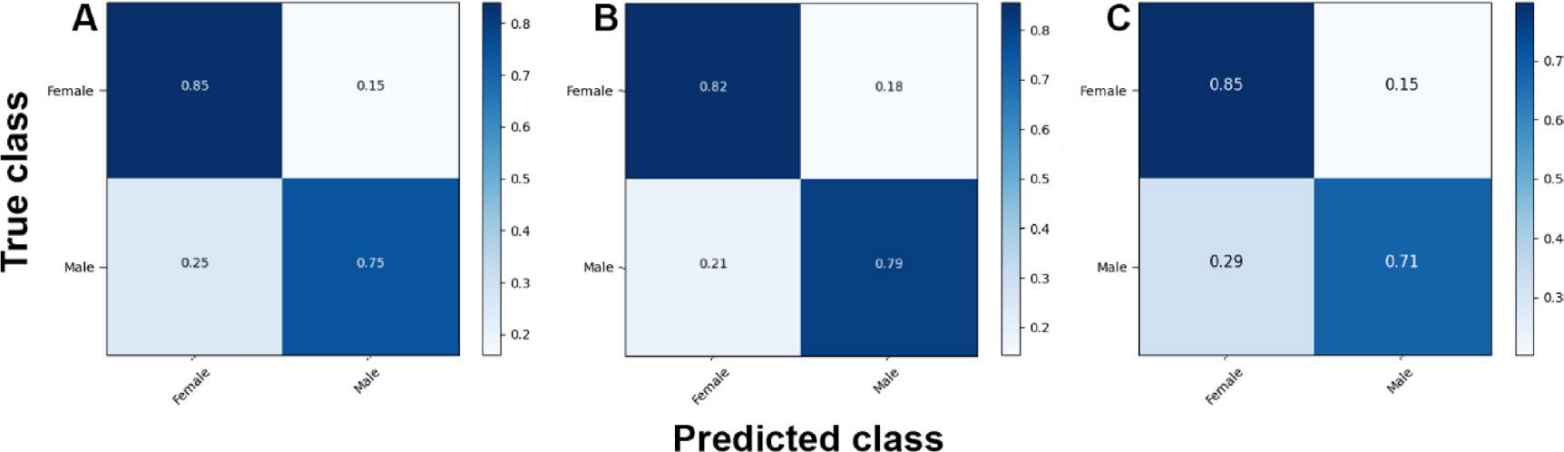
Confusion matrices showing the correct and incorrect classification rates based on sex for the three best performing models: DeiT (**A**), ViT (**B**) and EfficientNetV2M (**C**).

**Fig. 3:**
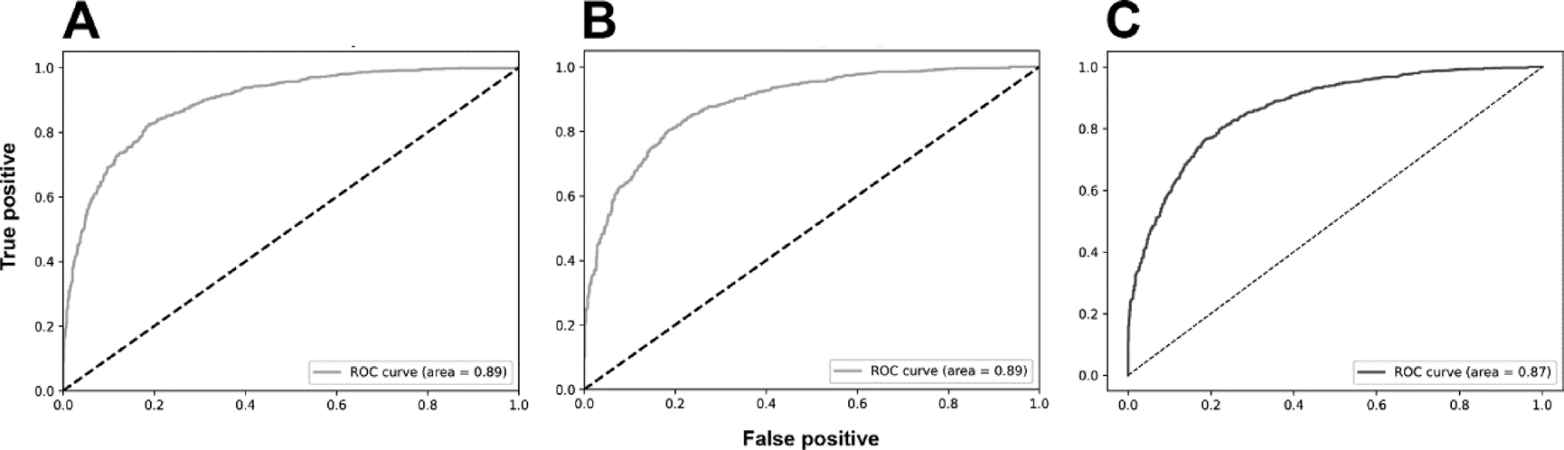
Receiver operating characteristic (ROC) curves and their respective area under the curve (AUC) for the three best performing models: DeiT (**A**), ViT (**B**) and EfficientNetV2M (**C**) after binary sex classification.

**Fig. 4:**
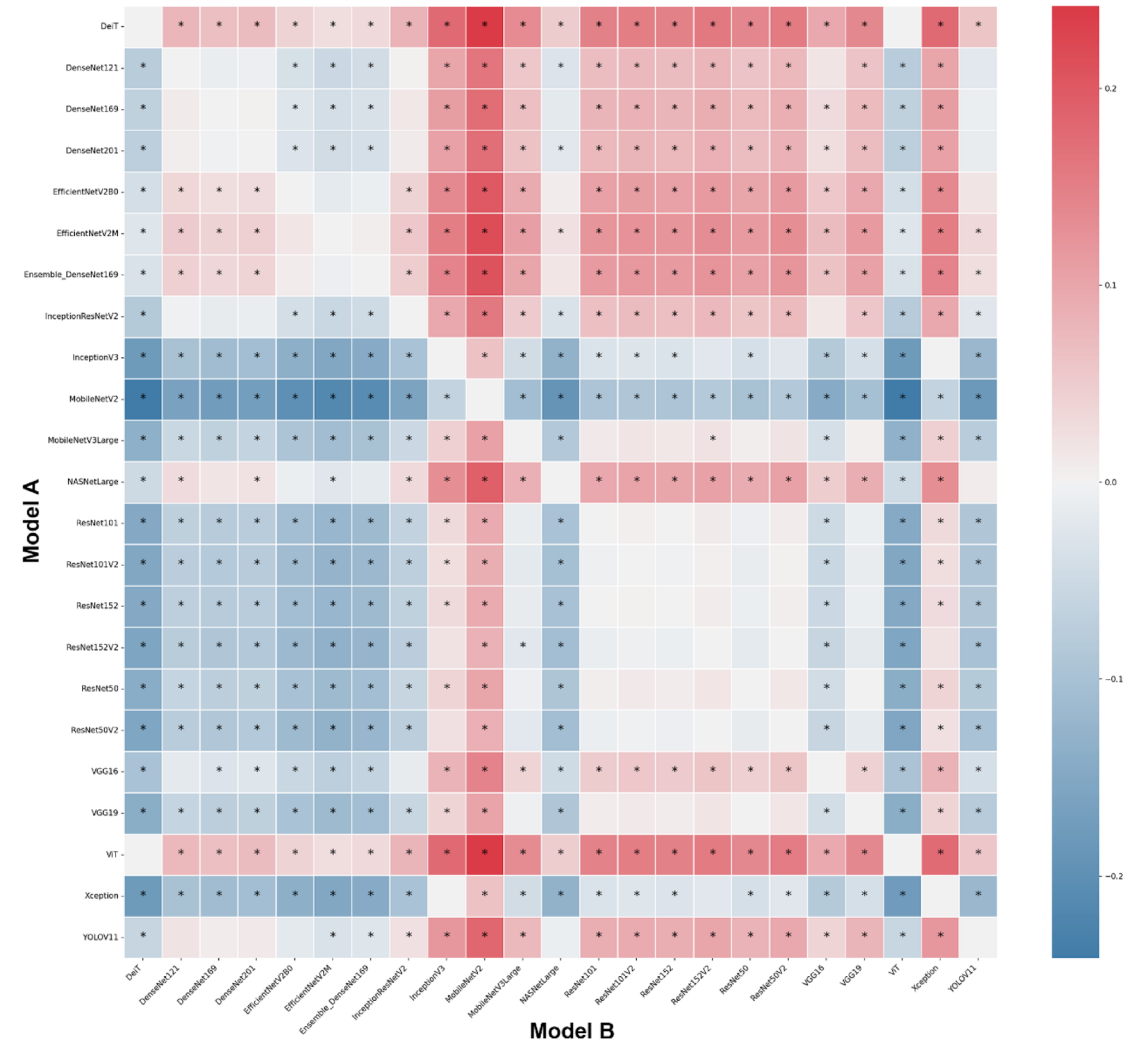
Heatmap of the pairwise accuracy differences between models illustrating the comparative performance based on sex classification with colors representing the magnitude and direction of accuracy differences. Statistical significance was assessed using bootstrap resampling (*p* < 0.05) and marked with an asterisk.

**Fig. 5:**
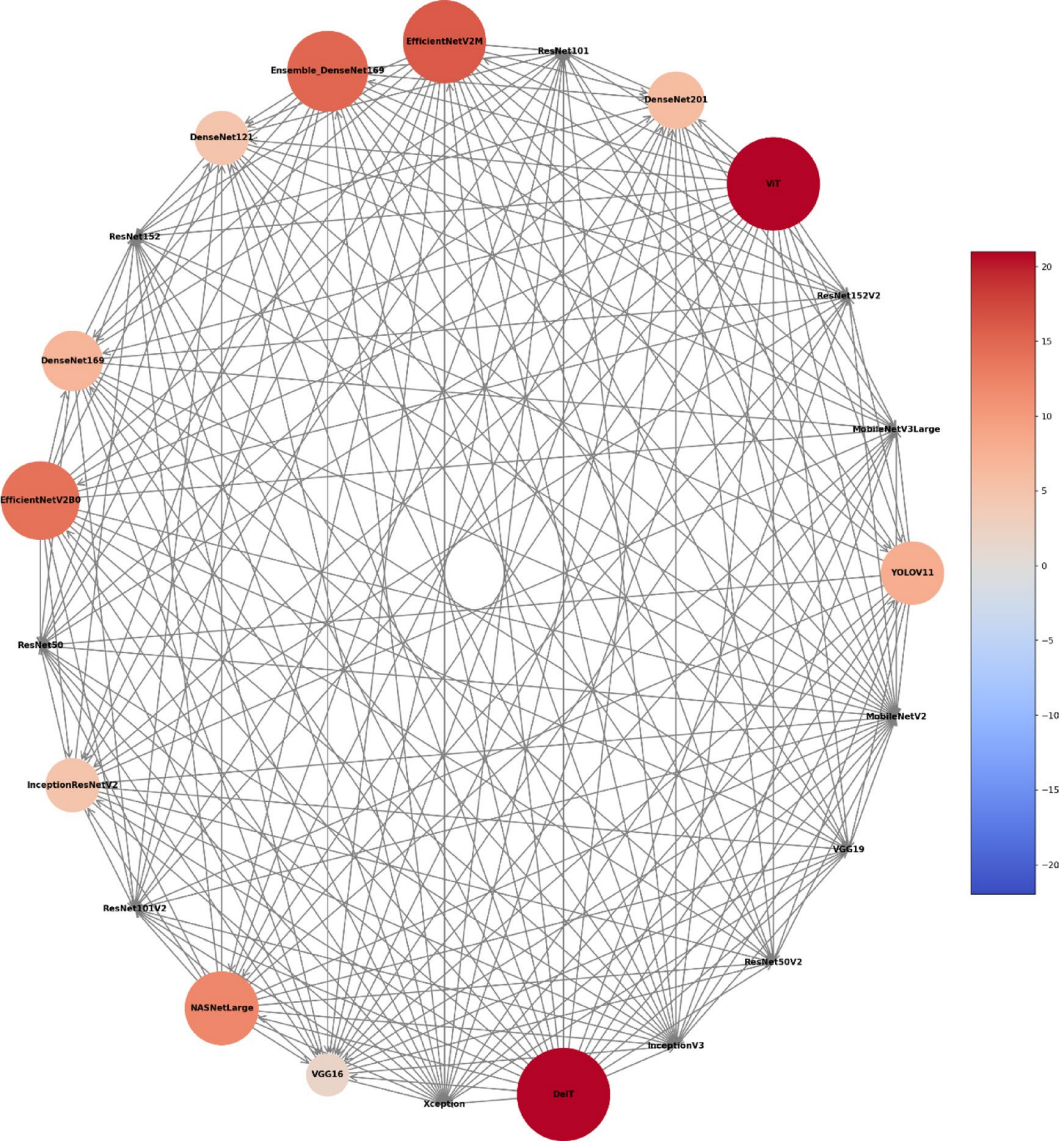
Circular network of model superiority displaying pairwise comparisons among models based on sex classification. Each node represents an individual model, with node size and colour proportional to the number of victories in direct comparisons against other models.

The analyses based on sex led to acc rates in the test phase between 0.565 and 0.807. The three best performing architectures based on acc were DeiT (acc = 0.807, CI95% 0.789; 0.824), followed by ViT (acc = 0.806, CI95% 0.789; 0.822) and EfficientNetV2M (acc = 0.781, CI95% 0.762; 0.799). For these architectures, F1-scores were 0.791 (CI95% 0.763; 0.805), 0.785 (CI95% 0.771; 0.809) and 0.751 (CI95% 0.728; 0.773), respectively. (Table 2). The correct classification rates of females were 85%, 82% and 85% for DeiT, ViT and EfficientNetV2M, respectively. In males, they were 75%, 79% and 71%, respectively (Fig. 2). For these architectures, the ROC curves were 0.89, 0.89 and 0.87, respectively (Fig. 3). When compared to the other 22 architectures, DeiT presented a superior performance with statistically significant differences (*p* < 0.05) against 21 of them (Fig. 4). The hierarchical superiority of the addressed architectures for sex classification is demonstrated in Fig. 5.

**Table 2:** Sex-classification task metrics for the performance of all the architectures addressed in the present study in the test and validation phases.

Architecture	Validation (K-folds average metrics)							Test					
	Epochs	Loss	Acc	F1	Precision	Recall	Specificity	Loss	Acc	F1	Precision	Recall	Specificity
DenseNet121	100	0.576	0.727	0.722	0.680	0.770	0.689	0.518	0.726	0.726	0.682	0.777	0.682
DenseNet169		0.550	0.746	0.729	0.711	0.748	0.745	0.529	0.738	0.714	0.729	0.698	0.773
DenseNet201		0.585	0.737	0.738	0.684	0.800	0.682	0.553	0.735	0.737	0.688	0.793	0.685
VGG16		0.571	0.712	0.679	0.687	0.671	0.746	0.544	0.709	0.680	0.700	0.661	0.752
VGG19		0.633	0.689	0.651	0.670	0.633	0.736	0.605	0.667	0.632	0.654	0.611	0.717
ResNet50		0.606	0.681	0.649	0.650	0.648	0.709	0.617	0.667	0.638	0.649	0.627	0.703
ResNet50V2		0.622	0.667	0.621	0.631	0.611	0.712	0.626	0.651	0.607	0.641	0.576	0.717
ResNet101		0.633	0.657	0.642	0.622	0.664	0.651	0.626	0.658	0.644	0.627	0.661	0.656
ResNet101V2		0.616	0.666	0.605	0.635	0.577	0.736	0.610	0.652	0.599	0.648	0.556	0.736
ResNet152		0.619	0.674	0.631	0.642	0.620	0.718	0.618	0.658	0.616	0.647	0.589	0.718
ResNet152V2		0.611	0.667	0.628	0.645	0.612	0.744	0.633	0.653	0.609	0.643	0.579	0.741
InceptionV3		0.651	0.628	0.524	0.589	0.472	0.747	0.651	0.628	0.551	0.633	0.488	0.752
Xception		0.648	0.632	0.576	0.600	0.546	0.705	0.627	0.628	0.577	0.616	0.543	0.703
InceptionResNetV2		0.688	0.748	0.732	0.714	0.750	0.746	0.722	0.725	0.712	0.698	0.726	0.725
NASNetLarge		0.555	0.754	0.727	0.741	0.713	0.788	0.505	0.757	0.735	0.750	0.720	0.790
MobileNetV2		0.681	0.577	0.535	0.536	0.533	0.613	0.679	0.565	0.528	0.535	0.522	0.603
MobileNetV3Large		0.614	0.682	0.677	0.658	0.697	0.669	0.605	0.668	0.660	0.633	0.689	0.650
EfficientNetV2B0		0.492	0.783	0.757	0.760	0.754	0.806	0.464	0.765	0.746	0.753	0.738	0.788
EfficientNetV2M		0.471	**0.792**	0.750	0.803	0.703	0.862	0.487	**0.781**	0.751	0.800	0.708	0.845
Ensemble		0.598	0.788	0.782	0.753	0.753	0.765	0.606	0.773	0.771	0.730	0.816	0.736
YOLOV11		0.550	0.772	0.751	0.768	0.734	0.804	0.562	0.748	0.723	0.744	0.703	0.788
ViT		0.698	**0.803**	0.780	0.794	0.767	0.833	0.668	**0.806**	0.791	0.796	0.785	0.824
DeiT		0.734	**0.807**	0.780	0.813	0.749	0.855	0.699	**0.807**	0.785	0.818	0.754	0.853

Acc: Accuracy. Ensemble: EfficientNetV2M-DenseNet169.

Bold meaning the highest accuracy (acc) values.

**Fig. 6:**
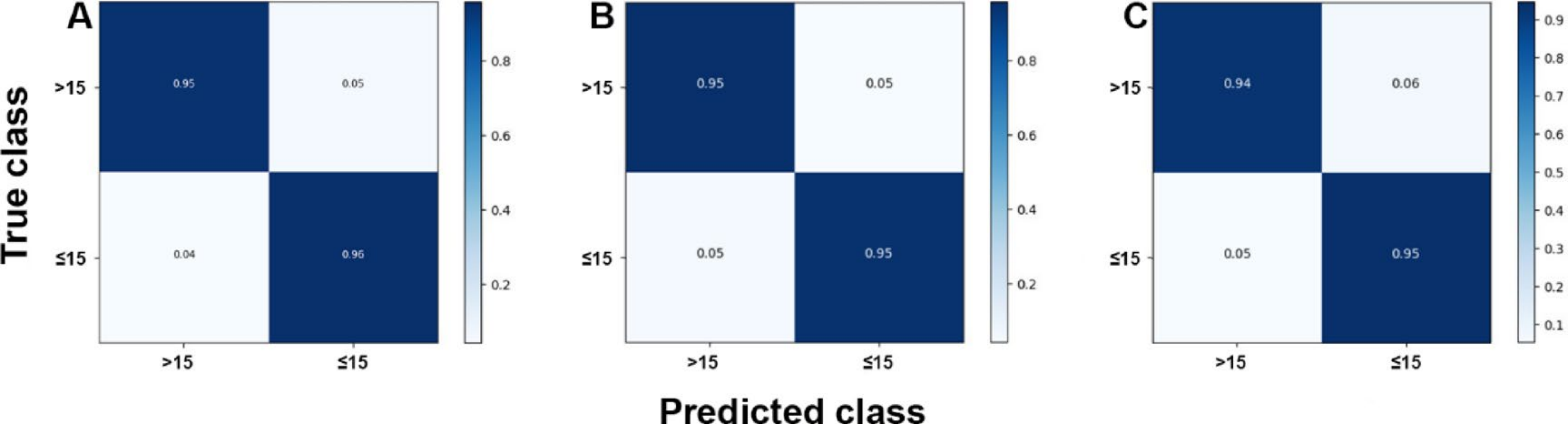
Confusion matrices showing the correct and incorrect classification rates based on age for the three best performing models: YOLOv11 (**A**), ViT (**B**) and DeiT (**C**).

**Fig. 7:**
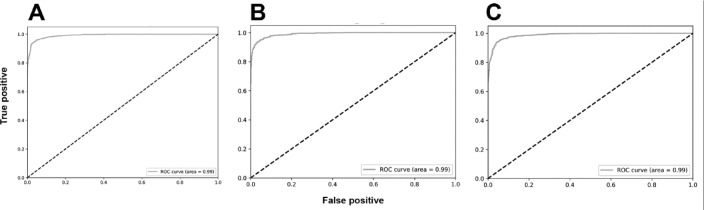
Receiver operating characteristic (ROC) curves and their respective area under the curve (AUC) for the three best performing models: YOLOv11 (**A**), ViT (**B**) and DeiT (**C**) after binary age classification.

**Fig. 8:**
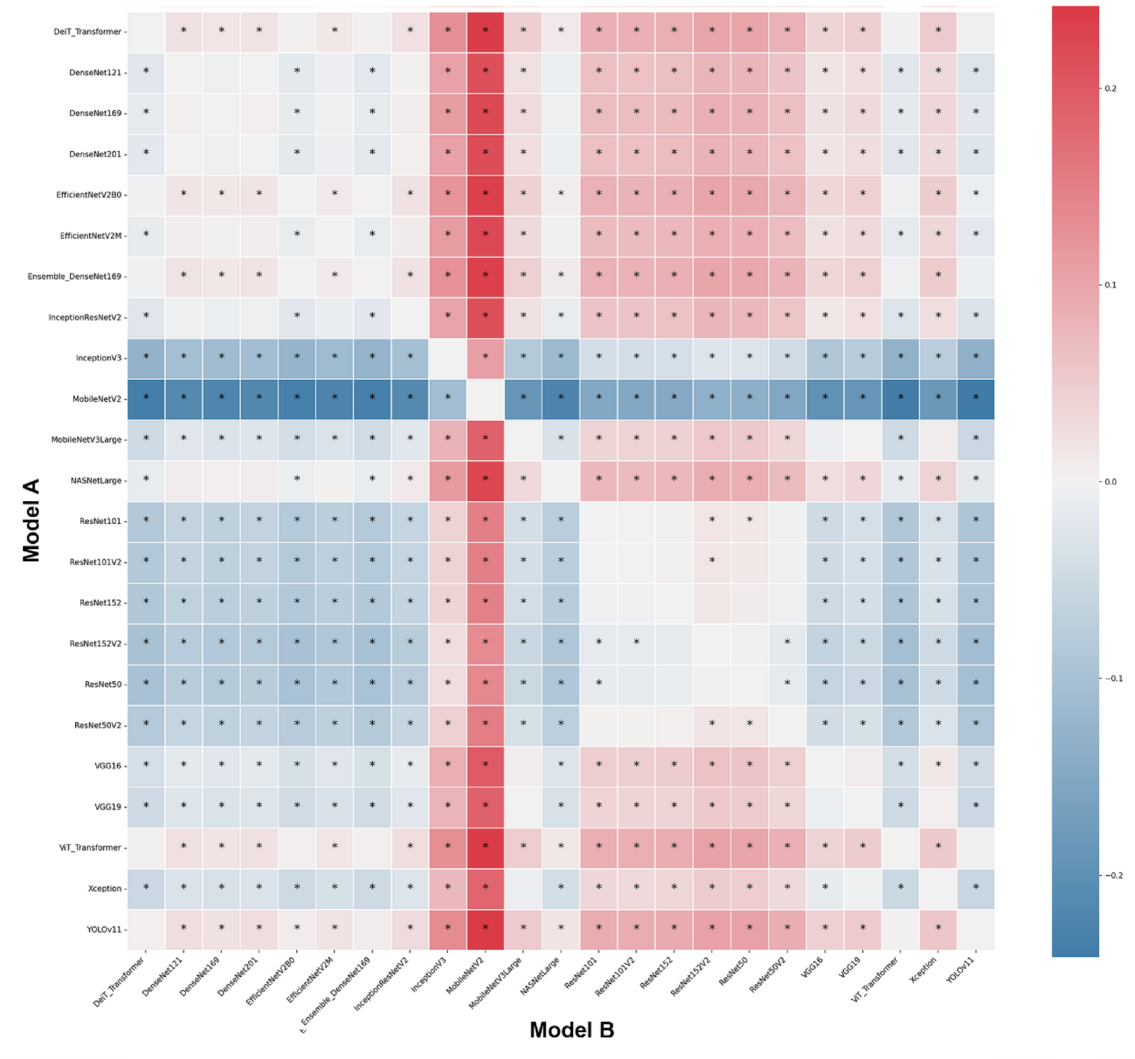
Heatmap of the pairwise accuracy differences between models illustrating the comparative performance based on age classification with colors representing the magnitude and direction of accuracy differences. Statistical significance was assessed using bootstrap resampling (*p* < 0.05) and marked with an asterisk.

**Fig. 9:**
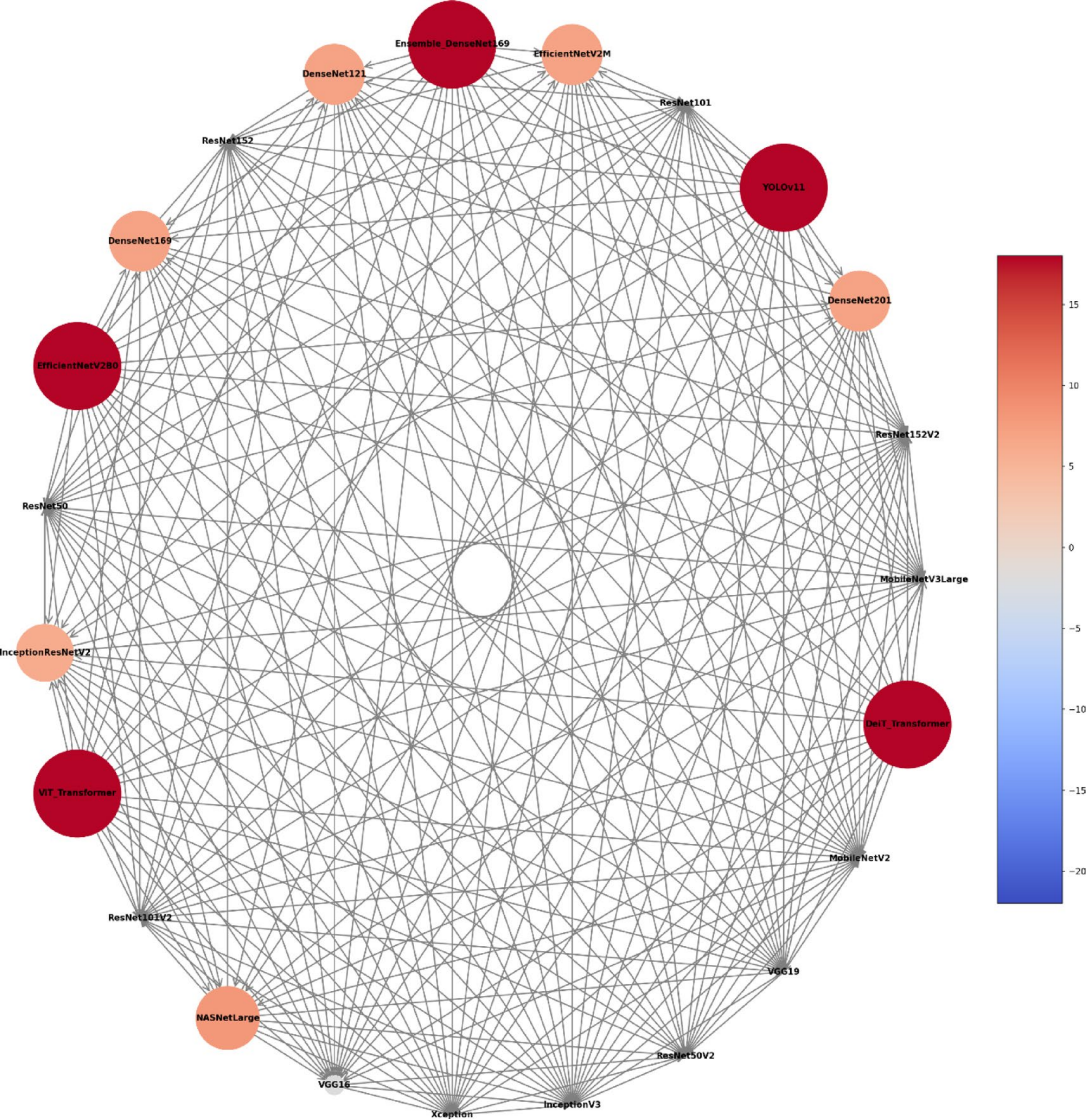
Circular network of model superiority displaying pairwise comparisons among models based on age classification. Each node represents an individual model, with node size and colour proportional to the number of victories in direct comparisons against other models.

The analysis based on age (≤ 15 and > 15 years) showed acc rates in the test phase between 0.650 and 0.953. The three best performing architectures based on acc were YOLOv11 (acc = 0.953, CI95% 0.944; 0.962), ViT (acc = 0.949, CI95% 0.940; 0.960) and DeiT (acc = 0.946, CI95% 0.936; 0.956). For these architectures, F1-scores were 0.953 (CI95% 0.947; 0.965), 0.952 (CI95% 0.943; 0.961) and 0.949 (CI95% 0.939; 0.959), respectively (Table 3). The correct classification rates of individuals ≤ 15 years were 96%, 95% and 95% for YOLOv11, ViT and DeiT, respectively. For individuals > 15 years, they were 95%, 95% and 94%, respectively (Fig. 6). All the ROC curves showed AUC of 0.99 (Fig. 7). Compared to the other architectures, YOLOv11, ViT and DeiT showed statistically significant differences (*p* < 0.05) against 19, 18 and 18 of them, respectively (Fig. 8). The hierarchical superiority of the addressed architectures for age classification is demonstrated in Fig. 9.

**Table 3:** Age-classification task metrics for the performance of all the architectures addressed in the present study in the test and validation phases.

Architecture	Validation (K-folds average metrics)							Test					
	Epochs	Loss	Acc	F1	Precision	Recall	Specificity	Loss	Acc	F1	Precision	Recall	Specificity
DenseNet121	100	0.220	0.915	0.921	0.942	0.901	0.933	0.209	0.920	0.924	0.930	0.918	0.922
DenseNet169		0.187	0.925	0.927	0.941	0.914	0.937	0.182	0.926	0.929	0.939	0.920	0.933
DenseNet201		0.196	0.927	0.927	0.944	0.911	0.944	0.184	0.921	0.923	0.955	0.893	0.953
VGG16		0.242	0.904	0.904	0.923	0.885	0.923	0.237	0.898	0.901	0.931	0.873	0.927
VGG19		0.250	0.898	0.900	0.908	0.891	0.904	0.258	0.888	0.892	0.907	0.878	0.899
ResNet50		0.367	0.840	0.837	0.891	0.789	0.894	0.404	0.823	0.824	0.870	0.783	0.868
ResNet50V2		0.340	0.085	0.849	0.892	0.809	0.896	0.360	0.843	0.843	0.897	0.794	0.897
ResNet101		0.399	0.840	0.830	0.903	0.768	0.915	0.403	0.868	0.835	0.908	0.772	0.912
ResNet101V2		0.332	0.859	0.856	0.927	0.796	0.929	0.325	0.842	0.840	0.910	0.779	0.913
ResNet152		0.355	0.852	0.849	0.923	0.786	0.926	0.388	0.836	0.831	0.915	0.761	0.920
ResNet152V2		0.384	0.839	0.823	0.949	0.727	0.958	0.398	0.820	0.808	0.935	0.741	0.944
InceptionV3		0.446	0.797	0.785	0.872	0.713	0.888	0.466	0.787	0.778	0.870	0.704	0.882
Xception		0.287	0.873	0.870	0.914	0.830	0.918	0.291	0.882	0.883	0.925	0.846	0.922
InceptionResNetV2		0.280	0.915	0.913	0.953	0.876	0.955	0.253	0.916	0.917	0.964	0.875	0.963
NASNetLarge		0.160	0.937	0.938	0.953	0.923	0.952	0.160	0.931	0.934	0.948	0.921	0.943
MobileNetV2		0.627	0.636	0.670	0.630	0.716	0.550	0.624	0.650	0.692	0.649	0.740	0.548
MobileNetV3Large		0.268	0.885	0.881	0.941	0.828	0.944	0.264	0.889	0.889	0.943	0.841	0.943
EfficientNetV2B0		0.156	0.939	0.940	0.945	0.936	0.942	0.132	0.943	0.946	0.956	0.955	0.952
EfficientNetV2M		0.175	0.938	0.941	0.932	0.951	0.924	0.189	0.929	0.933	0.925	0.941	0.914
Ensemble		0.156	0.941	0.942	0.946	0.946	0.944	0.130	0.944	0.947	0.948	0.945	0.942
YOLOV11		0.341	**0.979**	0.980	0.976	0.984	0.973	0.363	**0.953**	0.953	0.953	0.959	0.946
ViT		0.186	**0.950**	0.953	0.958	0.948	0.953	0.171	**0.949**	0.952	0.959	0.945	0.954
DeiT		0.140	**0.947**	0.951	0.946	0.956	0.937	0.144	**0.946**	0.949	0.946	0.953	0.938

Acc: Accuracy. Ensemble: EfficientNetV2M-DenseNet169. Bold meaning the highest accuracy (acc) values.

**Fig. 10:**
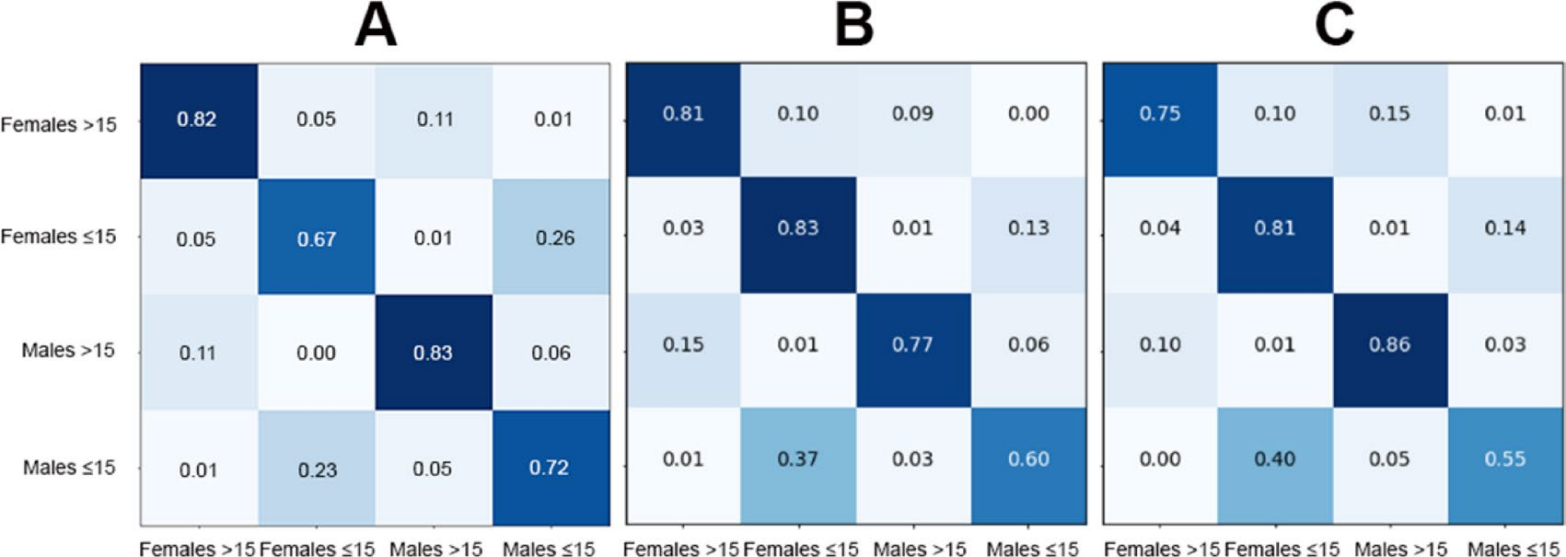
– Confusion matrices showing the correct and incorrect classification rates based on sex and age (multiclass) for the three best performing models: YOLOv11 (**A**), DeiT (**B**) and ViT (**C**).

**Fig. 11:**
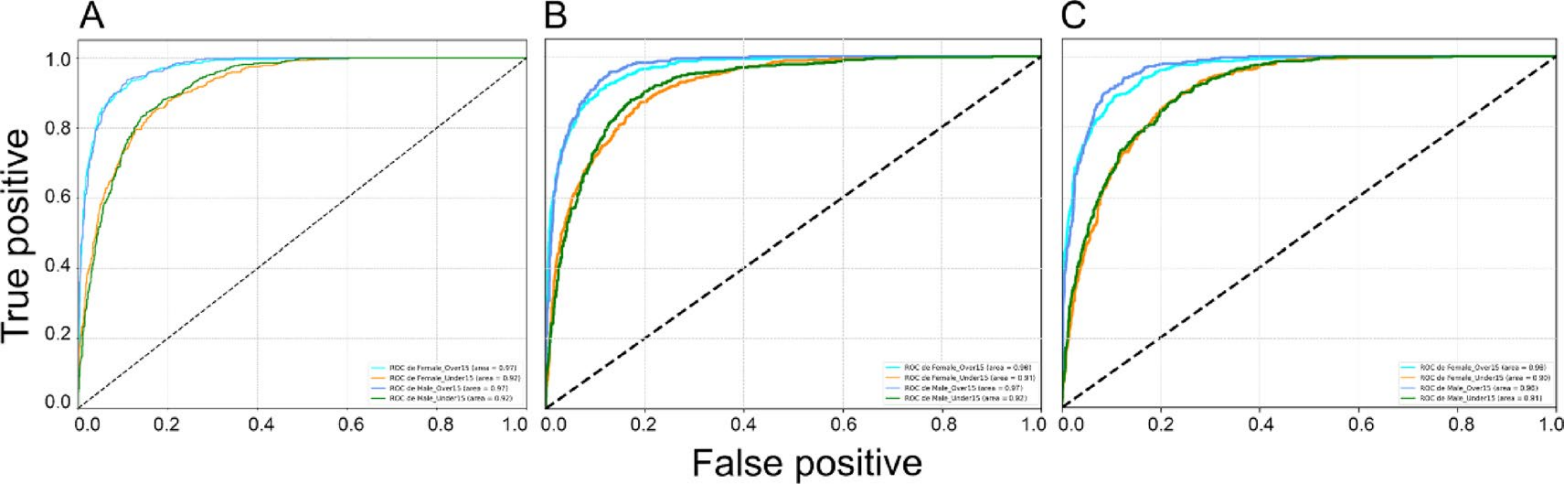
Receiver operating characteristic (ROC) curves and their respective area under the curve (AUC) for the three best performing models: YOLOv11 (**A**), DeiT (**B**) and ViT (**C**) after multiclass sex and age classification.

**Fig. 12:**
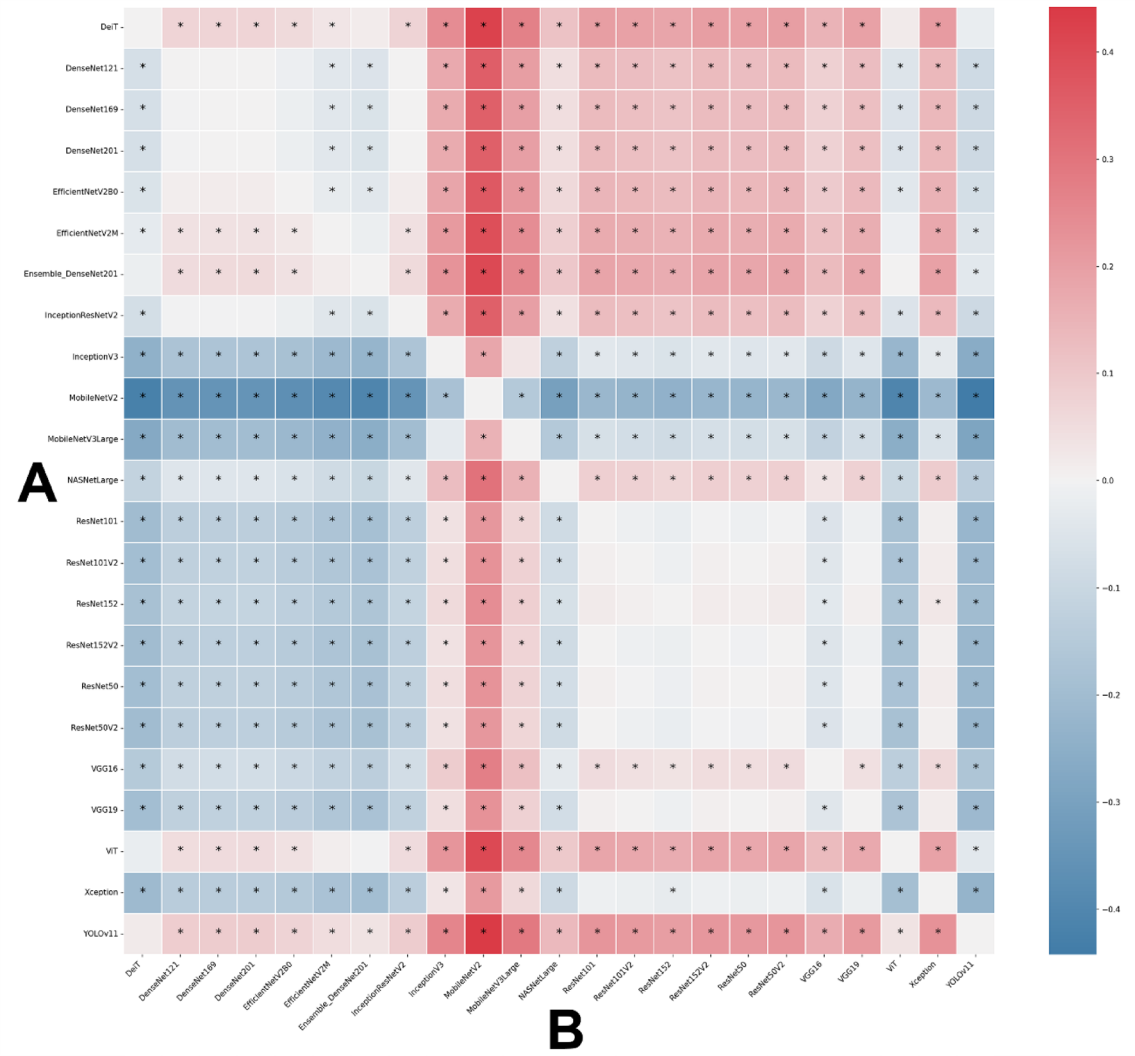
Heatmap of the pairwise accuracy differences between models illustrating the comparative performance based on sex and age classification with colors representing the magnitude and direction of accuracy differences. Statistical significance was assessed using bootstrap resampling (*p* < 0.05) and marked with an asterisk.

**Fig. 13:**
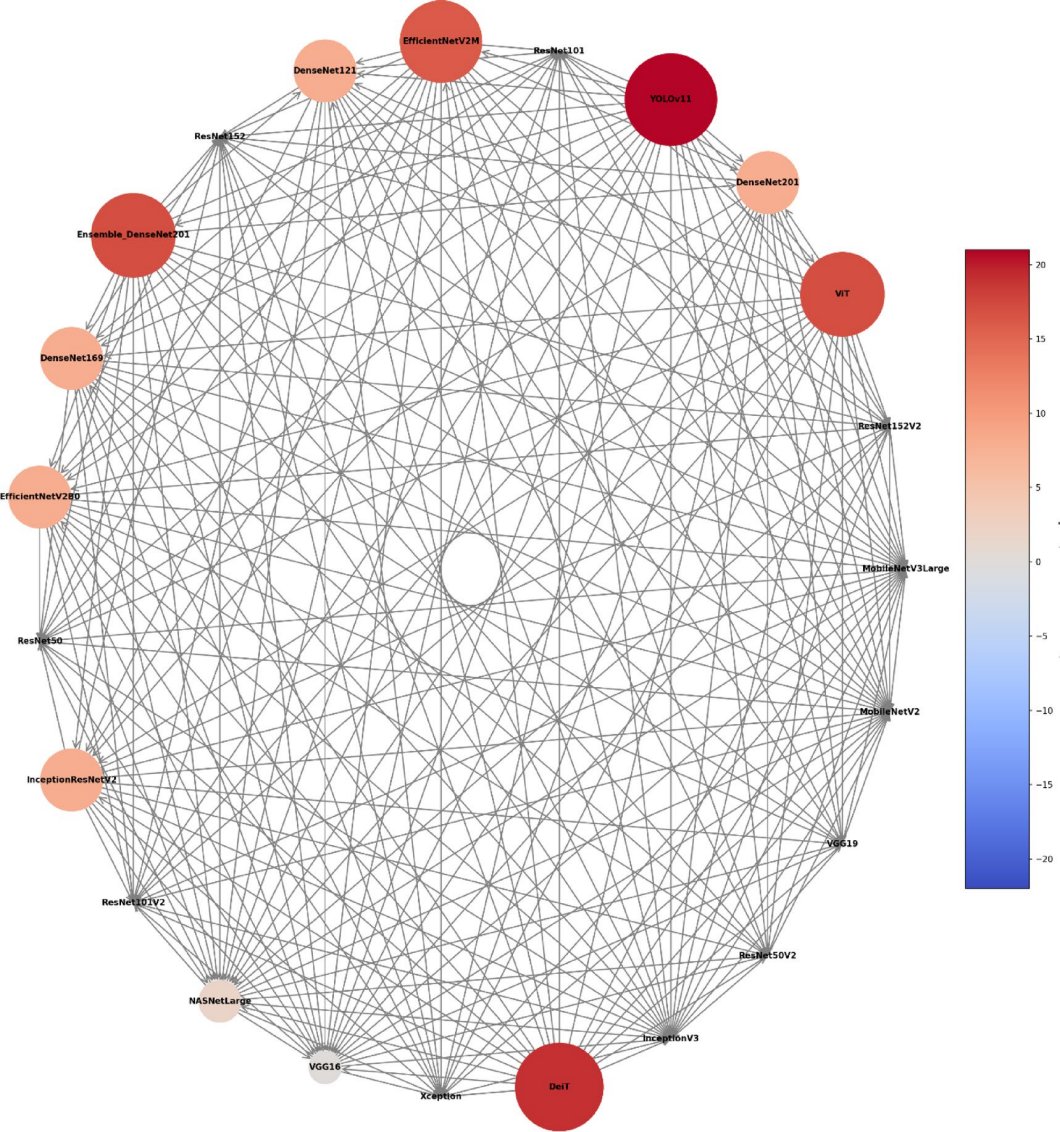
Circular network of model superiority displaying pairwise comparisons among models based on sex and age classification. Each node represents an individual model, with node size and colour proportional to the number of victories in direct comparisons against other models.

The multiclass analysis based on the combination of sex and age led to acc rates between 0.024 and 0.754. The best performing architectures based on acc were YOLOv11 (acc = 0.754, CI95% 0.752; 0.789), DeiT (*n* = 0.753, CI95% 0.734; 0.770) and ViT (acc = 0.734, CI95% 0.715; 0.753). For these architectures, F1-scores were 0.756 (CI95%: 0.753; 0.790), 0.732 (CI95% 0.736; 0.772) and 0.732 (CI95% 0.717; 0.754), respectively (Table 4). For YOLOv11, DeiT and ViT, the correct classification rates of females > 15 years was 82%, for 81% and 75%, while for females ≤ 15 years they were 67%, 83% and 81%, respectively. For males > 15 years, the correct classification rates were 83%, 77% and 86%, while for males ≤ 15 years they were 72%, 60%, and 55%, respectively (Fig. 10). ROC curves showed AUC of between 0.92 and 0.97 for YOLOv11, between 0.91 and 0.97 for DeiT and 0.90 and 0.96 for ViT (Fig. 11). YOLOv11 presented the best outcomes, showing statistically significant differences (*p* < 0.05) compared to 21 architectures, while DeiT and ViT statistically (*p* < 0.05) differed from 19 architectures (Fig. 12). Hierarchical superiority of the top three architectures based on the multiclass (sex and age) task was presented in Fig. 13.

**Table 4:** Multiclass sex-and-age-classification task metrics for the performance of all the architectures addressed in the present study in the test and validation phases.

Architecture	Validation (K-folds average metrics)							Test					
	Epochs	Loss	Acc	F1	Precision	Recall	Specificity	Loss	Acc	F1	Precision	Recall	Specificity
DenseNet121	100	0.755	0.686	0.681	0.705	0.705	0.908	0.778	0.685	0.677	0.697	0.659	0.904
DenseNet169		0.768	0.705	0.702	0.717	0.705	0.909	0.774	0.696	0.699	0.717	0.682	0.910
DenseNet201		0.744	0.725	0.722	0.736	0.715	0.915	0.745	0.705	0.696	0.710	0.682	0.907
VGG16		0.895	0.632	0.608	0.683	0.671	0.915	0.923	0.627	0.593	0.677	0.527	0.916
VGG19		0.889	0.608	0.578	0.668	0.658	0.915	0.918	0.571	0.537	0.627	0.470	0.907
ResNet50		0.958	0.577	0.539	0.630	0.625	0.907	0.977	0.572	0.540	0.634	0.470	0.909
ResNet50V2		0.961	0.575	0.497	0.643	0.625	0.925	0.995	0.548	0.476	0.638	0.379	0.928
ResNet101		0.927	0.581	0.565	0.619	0.595	0.893	1.010	0.547	0.535	0.588	0.491	0.885
ResNet101V2		0.931	0.586	0.555	0.634	0.602	0.904	0.961	0.545	0.529	0.607	0.469	0.898
ResNet152		0.933	0.603	0.561	0.643	0.625	0.907	1.006	0.584	0.545	0.636	0.477	0.909
ResNet152V2		0.948	0.575	0.549	0.629	0.592	0.904	0.996	0.545	0.522	0.610	0.456	0.902
InceptionV3		1.059	0.531	0.483	0.596	0.472	0.908	1.036	0.524	0.475	0.603	0.392	0.914
Xception		0.927	0.574	0.522	0.633	0.525	0.914	0.932	0.541	0.489	0.616	0.405	0.915
InceptionResNetV2		0.778	0.712	0.709	0.721	0.702	0.910	0.802	0.655	0.678	0.690	0.667	0.900
NASNetLarge		1.015	0.660	0.556	0.775	0.715	0.957	0.986	0.641	0.530	0.749	0.410	0.954
MobileNetV2		1.393	0.321	0.032	0.408	0.395	1.384	0.322	0.024	0.024	0.342	0.012	0.991
MobileNetV3Large		0.995	0.487	0.244	0.568	0.354	0.568	1.007	0.492	0.233	0.542	0.148	0.958
EfficientNetV2B0		0.660	0.730	0.727	0.737	0.680	0.914	0.718	0.711	0.706	0.716	0.696	0.908
EfficientNetV2M		0.858	0.749	0.750	0.752	0.715	0.918	1.005	0.721	0.723	0.728	0.718	0.910
Ensemble		0.933	0.760	0.761	0.763	0.624	0.921	1.017	0.750	0.749	0.751	0.747	0.917
YOLOV11		0.685	**0.872**	0.874	0.872	0.877	0.957	0.725	**0.754**	0.756	0.755	0.760	0.917
ViT		0.585	**0.754**	0.738	0.684	0.800	0.682	0.553	**0.734**	0.732	0.747	0.734	0.910
DeiT		0.906	**0.760**	0.727	0.741	0.713	0.788	0.923	**0.753**	0.752	0.763	0.753	0.916

Acc: Accuracy. Ensemble: EfficientNetV2M-DenseNet169.Bold meaning the highest accuracy (acc) values.

## Discussion

The MS are anatomic structures of interdisciplinary interest^61^. Studies with computer vision assessment of the MS have been performed for the diagnostic accuracy test of detecting sinusitis from panoramic radiographs applying transfer learning^62^. Authors have observed high performance of AI solutions binarily classifying the MS into labeled classes “healthy or inflamed”, and using developed source models trained with 350 images with subsequent inclusions of up to 225 radiographs^62^. In addition to the authors’ binary setting, they also employed a rectangular selection on region of interest over the MS. In our study two binary classifications were adopted, one for sex and the other for age, leading to moderate acc metrics in the former and high metrics in the latter.

For sex estimation the three best performing architectures showed acc rates around 80%. While on one hand this could be a promising outcome compared to part of the scientific literature that has detected lower acc rates^7,33^ on the other hand the present study’s sex-based outcome suggests that one in every five estimates would be incorrect. Translating to practice, an 80% acc rate could be high at first glance but not optimal to support the MS as sole features for sex estimation to forensic purposes. More specifically, authors^63^ have suggested that an acc of at least 80% is the minimum acceptable threshold for sex estimation methods based on dentomaxillofacial features. What dragged our outcomes close to the minimum acceptable could be the original sample age interval. To challenge the AI solutions with a more complex task, we have included children and young adolescents in the sample. The challenge relies on the fact that sexual dimorphism is less pronounced in children given the reduced expressions of endocrinological changes between boys and girls^64^. Consequently, the application of sex estimation methods is not recommended in individuals younger than 12 years of age^65^. In Brazil, the original location for the present study’s sample collection, the age of 12 years represents the legal transition from childhood to adolescence^66^. In practice, it means that in Brazilian territory it is preferrable to apply sex estimation methods in adults and, whenever technically feasible and scientifically reliable, adolescents. Hence, it is estimated that a sample fully composed of adults could increase the performance metrics of the studied models. Interestingly, within the top three performing models, the correct classification rates were higher among females (scores being 3–14% higher than males). Authors have stated that the volume of the MS can be more dispersed among males^67^, suggesting a higher morphological variability in this group. The position of dental roots, posterior tooth loss and developmental factors are examples of variables that may influence MS morphology leading, for instance, to size-related alterations, such as pneumatization. The latter has been reported as more pronounced in the young adulthood (18–34 years old) compared to older individuals^68^. Since the present study sampled individuals up to the age of 22.99 years, it is expected that some level of MS morphological variability can be introduced. In terms of computer vision, a higher within-group morphological variability can pose challenges to image pattern recognition, possibly being one of the reasons behind the more expressive difficulty of the models to correctly classify males. The sex-based outcomes observed in the first part of the present study corroborate the knowledge that age can not be ignored in the context of sex estimation^69^ – which leads us to the second part of our study.

When age-based analyses were conducted, higher performance metrics were observed. This research step, however, only consisted of a preliminary procedure before the third and final multiclass task. Because age should be kept in mind when planning sex estimation^69^, a methodological decision to split the sample below or above the age of 15 was adopted. This was because we aimed to assess the MS predictive potential in more (> 15 years) and less (≤ 15 years) developed individuals. By doing so, we observed optimal classification rates. It must be noted that, by dichotomizing the sample based on an age cutoff, classification performance is influenced by the relative distance of individuals from the decision threshold. In practical terms, individuals situated at the extremes of the age distribution are more easily classified. For instance, considering the present dataset ranging from 6 to 22.99 years and the division into two groups (≤ 15 years and > 15 years), the algorithm will tend to achieve higher acc when classifying younger individuals close to 6 or 7 years, compared to those near the cutoff (14–15 years). Similarly, within the older group, individuals at the upper end of the distribution can be more readily identified as belonging to the > 15 years category. This phenomenon indicates that the binary classification task may benefit from the clear separation provided by age extremes, which could inflate the overall acc rates. In this context, experts should not be blinded by the seemingly optimized performance of the studied models, but rather approach them with caution, considering the possibility of their use as adjunctive tools for age classification. In fact, for younger individuals, a wider and more reliable range of morphological parameters is available — most notably dental development^70,71^ — which should be preferred when the goal is age estimation in subjects under 15 years of age. This is because, with the exception of third molars, permanent teeth follow a developmental course that tends to be completed around 15–16 years of age^72^.

The multiclass assessment of sex and age combined yielded acc rates that were considerably lower, raging around 75% in test sample. This reduction in performance can be attributed to the higher complexity of the classification problem, as the model is required to simultaneously discriminate between multiple categories rather than a single dichotomous outcome. In the context of radiographic analysis, this challenge can be more evident, as subtle morphological variations related to sexual dimorphism and age progression may overlap between classes. While binary frameworks benefit from single decision thresholds (i.e. male or female, or ≤ 15 years and > 15 years), multiclass approaches must capture a broader spectrum of biologically related image features. Consequently, the multiclass task can deal with more variation within each group and less obvious separation between groups.

What should be highlighted is that across all three tasks, transformers models were always ranked among the three best performing architectures. Specifically, the best results emerged from DeiT^56^ and ViT^40^, which can be largely attributed to their self-attention mechanisms, allowing them to capture long-range dependencies and global contextual patterns in the entire image. This contrasts with conventional CNNs, which can be useful to detect localized features, but may miss broader structural relationships that can be abundant in panoramic radiographs. Other architectures also performed consistently, such as YOLOv11^55^ and EfficientNetV2M^54^. The first, represents one of the most advanced detection frameworks, capable of extracting highly representative features and integrating them efficiently, while the second balances network depth, width, and resolution through compound scaling and optimized training, allowing it to learn powerful features while keeping computational costs reasonable. These models illustrate how newer architectures that emphasize global context (Transformers)^40,56^ or efficient scaling and detection (YOLOv11 and EfficientNetV2M)^54,55^ provide an advantage over traditional CNNs when handling the subtle and distributed features found in panoramic radiographs.

Comparisons between architectures also have been reported in recent scientific literature. Alam et al. (2025)^73^ conducted a small-scale investigation (*n* = 437) evaluating multiple neural network models (VGG16, VGG19, ResNet50, ResNet101, ResNet152, MobileNet, DenseNet121, DenseNet169) alongside Vision Transformer architectures for simultaneous sex and age estimation from panoramic radiographs. While their design shares conceptual similarity with our approach, the markedly limited sample size constrains generalizability and statistical power. Moreover, their dataset consisted predominantly of adult individuals, a group in which dentomaxillofacial structures are largely stabilized; in such cases, chronological inference becomes increasingly dependent on regressive rather than progressive morphological parameters for age estimation, diminishing direct association to age-related variation. Their results showed the best performance for DenseNet169, highlighting CNNs’ efficiency. In contrast, our substantially larger dataset spanning a broad developmental range of young individuals—allowed the evaluation of modern architectures. Our results demonstrated superior performance for architectures that are not strictly convolutional. These consistently outperformed CNNs, suggesting that transformer-based attention mechanisms and hybrid feature representations may generalize better across diverse morphologic and developmental contexts captured in panoramic radiography – at least for analyses based on MS. Complementarily, Wang et al. (2023)^74^ compared VGG16 and ResNet101 for dental age estimation in 9,586 panoramic radiographs (4054 males and 5532 females aged 6–20 years). Their results confirmed VGG16’s superiority, achieving an acc of 93.6% in the 6–8 year category, supporting the notion that dental age prediction is most reliable in younger individuals due to the presence of concurrent developmental markers. Unlike their categorical multi-age framework (6–8, 9–11, 12–14, 15–17, 18–20 years), our binary age classification design simplified the estimation process, while maintaining some level of adjuvant applicability for forensic purposes. When it comes to VGG16’s performance, this architecture has not appeared in the top performing models of the present study in any task, suggesting that it could be a better tool for dental age estimation than for age-related MS assessment.

This work is not exempt from limitations. One of the most frequent concerns in the field is the choice of two-dimensional radiographs instead of computed tomography. Although tomographic images offers superior anatomical detail, its use can be constrained by high operational costs and the considerable computational power required to process volumetric datasets in virtual environments, which limits its feasibility in large-scale studies. Moreover, panoramic radiographs can be sensitive to variations in equipment type, acquisition protocols, detector technology, and patient positioning, all of which can alter image quality and anatomical visibility of the MS. These factors may affect the stability of AI feature extraction and, consequently, the model’s generalization to external datasets. Furthermore, the presence of sinus-related alterations, such as mucosal thickening, retention cysts, and other opacifications, can modify the apparent morphology and radiodensity of the sinus walls, hampering the detection of the MS’ anatomical outlines. Hence, model robustness across different acquisition protocols remains to be confirmed. Future multicentric validations encompassing a wider range of radiographic machines, exposure parameters, and clinical conditions are essential to ensure that the proposed AI models maintain consistent performance in diverse real-world scenarios. Another important avenue for future research is the design of more challenging classification tasks, such as the use of narrower age intervals. This strategy would reduce the bias introduced by individuals positioned at the extremes of broader categories, where classification tends to be artificially facilitated by the distance from the decision threshold. The present study was not designed to evaluate model performance across refined age categories (e.g., one-year intervals such as 6–6.9, 7–7.9, …, 22–22.99 years). Implementing such an approach would require a considerably larger dataset to ensure adequate representation, generalization, and statistical robustness. Although this type of fine-grained analysis could provide valuable insights, it was not feasible within the constraints of the available sample. The dataset was balanced per sex and one-year age categories, serving primarily as a quality control step to achieve a more uniform distribution rather than enabling detailed age estimation from the MS. Consequently, a binary age classification framework was adopted instead of generating predictions based on a more stratified categorical variable. This approach provided a more stable assessment of model performance while minimizing the effects of limited sample size and ensuring that the results remained interpretable within the study’s scope.

By incorporating advanced imaging modalities when feasible, and by refining experimental designs to minimize bias, future investigations could provide a more accurate assessment of the true potential and boundaries of artificial intelligence in sex and age estimation.

## Conclusion

This study demonstrated that Transformer-based architectures, particularly DeiT and ViT, consistently achieved superior performance in sex and age classification tasks using MS images annotated from panoramic radiographs. Modern CNN-based models such as YOLOv11 and EfficientNetV2M also ranked among the top performers, highlighting the potential of newer deep learning solutions to handle subtle and spatially distributed features more effectively than conventional CNNs.

Application of the high-ranked studied models for binary forensic sex and age classification is promising but should currently be regarded as complementary and not as an immediate replacement for existing solutions. Multiclass approaches, although encouraging, remain methodologically complex and less reliable for casework at this stage, especially considering the current study’s methodological settings.

## Supplementary Information

Below is the link to the electronic supplementary material.

Supplementary Material 1

## Data Availability

The data supporting this study’s findings are available from the project supervisor, Prof. Ademir Franco, upon reasonable request and with permission from the Center of Oral Radiology and Imaging.
