# Supplementary material for: Maxillary sinus classification for sex and age using 23 artificial intelligence architectures
Source: Sci Rep. 2026 Jan 19;16:5716. doi: 10.1038/s41598-026-36112-1 (PMC12891728; doi:10.1038/s41598-026-36112-1)
Supplement: Supplementary file 1 — Supplementary Material 1 [file 41598_2026_36112_MOESM1_ESM.docx]

| Section and topic | No. | STARD-AI item | Page |
| --- | --- | --- | --- |
| Title or abstract | 1 | Identification as a study reporting AI-centered diagnostic accuracy and reporting at least one measure of accuracy within title or abstract | 1 |
| Abstract | 2 | Structured summary of study design, methods, results and conclusions (see STARD for Abstracts) | 2 |
| Introduction | 3 | Scientific and clinical background, including the intended use of the index test, whether it is novel or established, and its integration into an existing workflow, if applicable | 3,4 |
|  | 4 | Study objectives and hypotheses | 5 |
| Study design | 5 | Whether data collection was planned before the index test and reference standard were performed (prospective study) or after (retrospective study) | 5 |
| Ethics | 6* | Formal approval from an ethics committee. If not required, justify why. | 5 |
| Participants | 7 | Eligibility criteria: listing separate inclusion and exclusion criteria in the order that they are applied at both participant level and data level | 6 |
|  | 8 | On what basis potentially eligible participants were identified (such as symptoms, results from previous tests and inclusion in registry) | 6 |
|  | 9 | Where and when potentially eligible participants were identified (setting, location and dates) | 6 |
|  | 10 | Whether participants formed a consecutive, random or convenience series | 6 |
| Dataset | 11* | Source of the data and whether they have been routinely collected, specifically collected for the purpose of the study or acquired from a third party repository | 6 |
|  | 12* | Who undertook the annotations for the dataset (including experience levels and background) and how (within the same clinical context or in a post hoc fashion), if applicable | 7 |
|  | 13* | Devices (manufacturer and model) that were used to capture data, software (with version number) used to engineer the index test, and details of the reference standard if applicable | 7 |
|  | 14* | Data acquisition protocols (for example, contrast protocol or reconstruction method for medical images) and details of data pre-processing, including any data normalization or augmentation | 7,8 |
| Test methods | 15a | Index test, in sufficient detail to allow replication | 9 |
|  | 15b | How the index test was developed, including any training, validation, testing and external evaluation, detailing sample sizes, when applicable | 9,10 |
|  | 15c | Definition of and rationale for test positivity cutoffs or result categories of the index test, distinguishing prespecified from exploratory | 9 |
|  | 15d* | The specified end-user of the index test and the level of expertise required of users | n/a |
|  | 16a | Reference standard, in sufficient detail to allow replication | 9 |
|  | 16b | Rationale for choosing the reference standard (if alternatives exist) | 9 |
|  | 16c | Definition of and rationale for test positivity cutoffs or result categories of the reference standard, distinguishing prespecified from exploratory | 9 |
|  | 17a | Whether clinical information and reference standard results were available to the performers or readers of the index test | n/a |
|  | 17b | Whether clinical information and index test results were available to the assessors of the reference standard | n/a |
| Analysis | 18 | Methods for estimating or comparing measures of diagnostic accuracy | 10 |
|  | 19 | How indeterminate index test or reference standard results were handled | 10 |
|  | 20 | How missing data on the index test and reference standard were handled | n/a |
|  | 21 | Any analyses of variability in diagnostic accuracy, distinguishing prespecified from exploratory | 10 |
|  | 22 | Intended sample size and how it was determined | n/a |
|  | 23* | Details of any performance error analysis and algorithmic bias and fairness assessments, if undertaken | n/a |
| Results |  |  |  |
| Participants and dataset | 24 | Flow of participants, using a diagram | n/a |
|  | 25’ | Baseline demographic, clinical and technical characteristics of training, validation and test sets, if applicable | 9 |
|  | 26a | Distribution of severity of disease in those with the target condition | n/a |
|  | 26b | Distribution of alternative diagnoses in those without the target condition | n/a |
|  | 27 | Time interval and any clinical interventions between index test and reference standard | n/a |
|  | 28* | Whether the datasets represent the distribution of the target condition that one would expect from the intended use population | n/a |
|  | 29* | For external evaluation on an independent dataset, an assessment of how this differs from the training, validation and test sets | 11 |
| Test results | 30 | Cross-tabulation of the index test results (or their distribution) by the results of the reference standard | 11 |
|  | 31 | Estimates of diagnostic accuracy and their precision (such as 95% confidence intervals) | 11 |
|  | 32 | Any adverse events from performing the index test or the reference standard | n/a |
| Discussion | 33 | Study limitations, including sources of potential bias, statistical uncertainty and generalizability | 16,17 |
|  | 34 | Implications for practice, including the intended use and clinical role of the index test | 12 |
|  | 35* | Ethical considerations and adherence to ethical standards associated with the use of the index test and issues of fairness | 13-15 |
| Other information | 36 | Registration number and name of registry | n/a |
|  | 37 | Where the full study protocol can be accessed | n/a |
|  | 38 | Sources of funding and other support, role of funders | Journal’s system |
|  | 39 | Commercial interests, if applicable | None |
|  | 40a* | Availability of datasets and code, detailing any restrictions on their reuse and repurposing | Journal’s system |
|  | 40b* | Whether outputs are stored, auditable and available for evaluation, if necessary | Journal’s system |
